# The resurgence of Lassa fever in Nigeria: economic impact, challenges, and strategic public health interventions

**DOI:** 10.3389/fpubh.2025.1574459

**Published:** 2025-07-16

**Authors:** Stanley Chinedu Eneh, Chidera Gabriel Obi, Udokang Ephraim Ikpongifono, Zakariya’u Dauda, Samson Adiaetok Udoewah, Collins Chibueze Anokwuru, Francisca Ogochukwu Onukansi, Ogechi Vinaprisca Ikhuoria, Temitope Olumuyiwa Ojo, Chukwuocha Uchechukwu Madukaku, Ibuchukwu Nkeonyenasoya Orabueze, Amara Frances Chizoba

**Affiliations:** ^1^Department of Community Health, Obafemi Awolowo University, Ile-Ife, Nigeria; ^2^IVAN Research Institute, University of Nigeria, Enugu, Nigeria; ^3^Youth in Research Hub, Enugu, Nigeria; ^4^Department of Parasitology and Entomology, Nnamdi Azikiwe University, Awka, Nigeria; ^5^Faculty of Pharmacy, University of Uyo, Uyo, Nigeria; ^6^School of Medical Laboratory Science, Usmanu Danfodiyo University Sokoto, Sokoto, Nigeria; ^7^Department of Public Health Nursing, Africa Centre of Excellence in Public Health and Toxicological Research, University of Port Harcourt, Port Harcourt, Nigeria; ^8^Department of Public Health, Federal University of Technology Owerri, Owerri, Nigeria; ^9^School of Public Health, University of Port Harcourt, Port Harcourt, Nigeria; ^10^Innovations and Technologies for Disease Control Research Group, Department of Public Health, Federal University of Technology, Owerri, Nigeria; ^11^Department of Medical Microbiology, Faculty of Basic Clinical Sciences, College of Medicine, University of Nigeria, Enugu, Nigeria; ^12^Department of Microbiology, University of Nigeria Teaching Hospital, Enugu, Nigeria; ^13^Mission to Elderlies Foundation, Anambra, Nigeria

**Keywords:** Lassa fever, outbreak, Nigeria, economic impact, re-emerging diseases, disease control, public health responses, One Health approach

## Abstract

Lassa fever remains a persistent public health challenge in Nigeria, with annual outbreaks expanding across the country. Between 2018 and 2023, the disease spread from 20 to 34 of Nigeria’s 37 states, underscoring its endemic nature. Recent data from the Nigeria Centre for Disease Control and Prevention (NCDC) indicates 1,171 suspected cases, 290 confirmed cases, and 53 deaths between January 6 to 26, 2025, with a case fatality rate of 18.3%. Lassa fever transmission is highly seasonal, peaking during the dry months when food scarcity drives rodent-human interactions. Inadequate early detection, weak surveillance systems, and economic constraints exacerbate the burden on Nigeria’s healthcare infrastructure. Environmental, socioeconomic, and systemic healthcare limitations drive the resurgence of Lassa fever. Climate change-induced shifts in temperature and precipitation patterns have disrupted rodent habitats, increasing human exposure to the virus. Additionally, poverty, poor sanitation, and urban expansion facilitate the proliferation of disease-carrying rodents. Limited funding and insufficient healthcare facilities hinder timely responses, contributing to high mortality rates. The economic impact extends beyond healthcare costs to agricultural disruptions, trade restrictions, and workforce productivity losses. To mitigate future outbreaks, Nigeria must adopt a multifaceted strategy that includes robust disease surveillance, the use of environmental data, effective rodent control measures, improved waste management, and strengthened cross-sectoral collaboration and policy implementation. Strengthening healthcare infrastructure, investing in vaccine development will enhance early detection and response efforts. By adopting an integrated One Health approach, Nigeria can improve disease control, reduce fatalities, and alleviate the economic burden of Lassa fever outbreaks.

## Introduction

Lassa fever has emerged as a persistent public health threat in Nigeria, with annual outbreaks reported since its discovery over 50 years ago. Initially restricted to a few regions, the disease has now spread to 34 of the country’s 37 states, affecting over 17% of Nigeria’s 774 local government areas (LGAs) (1–3). In 2025 alone, the Nigeria Centre for Disease Control and Prevention (NCDC) reported 1,171 suspected cases and 290 confirmed cases, with 53 deaths, resulting in a case fatality ratio (CFR) of 18.3% across 54 LGAs in 7 states (3). This expanding geographic spread and persistently high CFR emphasize Lassa fever’s endemic nature and the continued challenge it poses to Nigeria’s health system. Transmission is largely seasonal, peaking during the dry season (November to March) when natural food sources are scarce, encouraging rodents, the primary reservoirs, to seek food in human settlements (4). Public health systems must remain particularly vigilant during these periods. The fatality rate of Lassa fever in Nigeria currently ranges from 13.5 to 18.3% among confirmed cases, underscoring limitations in early detection, treatment, and public awareness (5). Despite ongoing surveillance efforts, the persistence and geographic expansion of Lassa fever, coupled with high fatality rates, underscore a critical gap in Nigeria’s preparedness and response strategies. There is limited data on the intersection of its economic burden and public health response effectiveness, especially in the context of recent outbreaks.

In addition to the public health burden, Lassa fever imposes substantial economic costs on Nigeria. The health sector bears the financial strain of managing isolation centers, intensive care units, diagnostic tools, medications, and personal protective equipment (PPE) (6, 7). Beyond healthcare, the disease disrupts other sectors, including agriculture, by destabilizing food supply chains, international trade, through export restrictions from affected regions, and education, via school closures in outbreak zones (6). These consequences are often intensified by underfunded health systems, limited access to effective treatments, and insufficient ownership at the subnational level (8). Collectively, these factors highlight Nigeria’s struggle to mitigate the burden of Lassa fever, particularly its economic implications.

**Figure 1:**
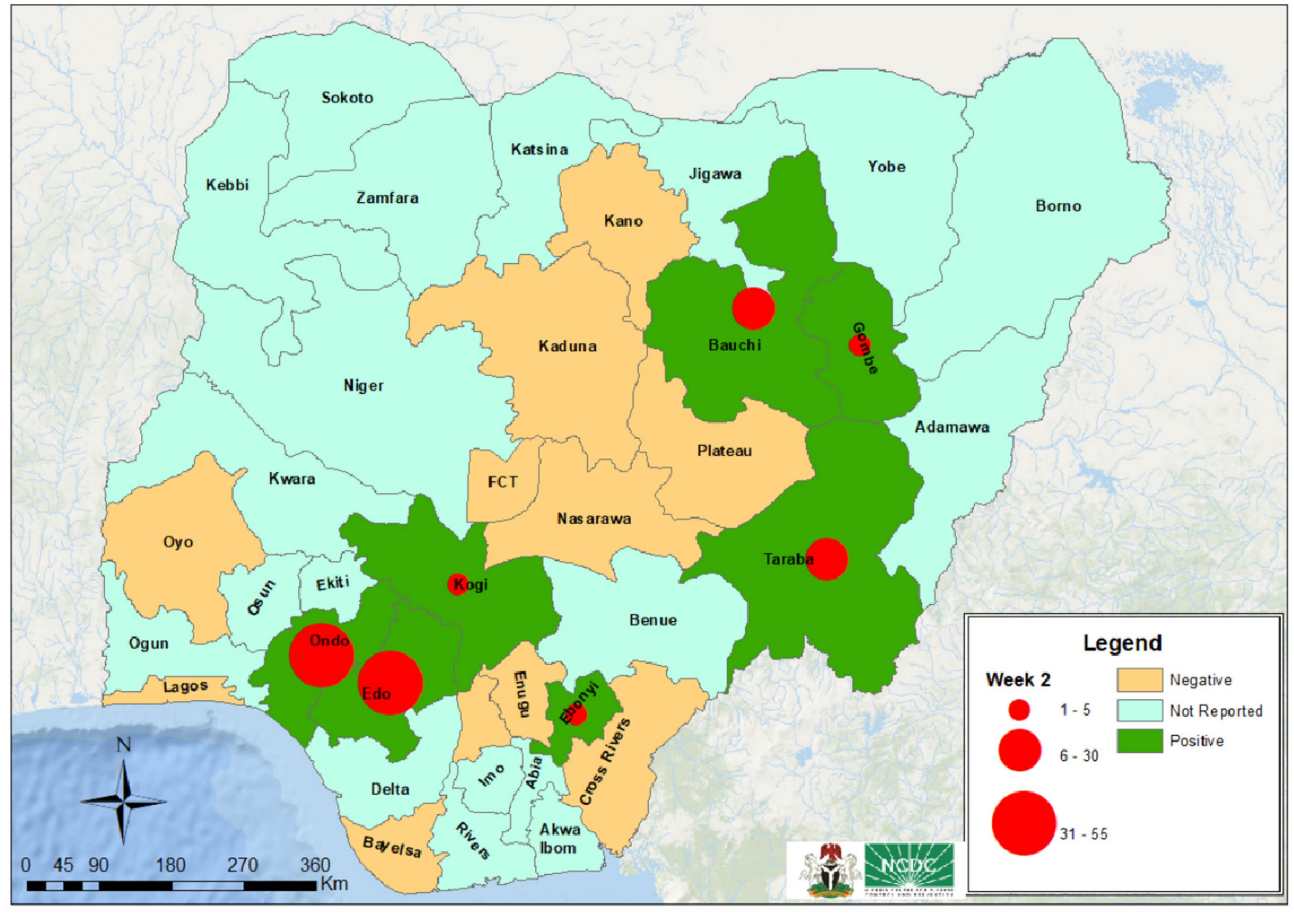
The most affected states with reported cases of Lassa fever (3).

Unlike previous studies that focus predominantly on the clinical or epidemiological aspects of Lassa fever (1, 9), this paper introduces a novel interdisciplinary framework that integrates real-time surveillance data with an analysis of the economic impact to evaluate and strengthen Nigeria’s current outbreak preparedness strategies. By combining public health interventions with insights from health economics and systems analysis, the study identifies structural weaknesses in subnational response capacity. This approach moves beyond descriptive epidemiology to offer actionable, data-informed strategies that can improve cholera outbreak, resource planning, and cross-sectoral resilience. This study describes the resurgence of Lassa fever in Nigeria, explores its socio-economic and public health implications, and proposes evidence-based recommendations to strengthen outbreak preparedness and control strategies (see Figure 1).

### Current outbreak: overview of Lassa fever outbreak in Nigerian states

According to the latest report from the NCDC, between January 6 to 26, 2025, new confirmed cases, suspected cases, and fatalities due to Lassa fever were reported in Ondo, Edo, Taraba, Bauchi, Gombe, Kogi, and Ebonyi states etc. According to the current reports from NCDC (3), in Southwestern Nigeria, Ondo State recorded 328 suspected cases, with 107 confirmed cases and 10 deaths among the confirmed cases. Edo State reported 358 suspected cases, with 61 confirmed cases and 10 deaths from the confirmed cases. In North-Eastern Nigeria, Bauchi State recorded 205 suspected cases, 49 confirmed cases, and 5 confirmed deaths (3). Taraba State had 104 suspected cases, 48 confirmed cases, and 15 deaths, while Gombe State reported 18 suspected cases, 5 confirmed cases, and 4 confirmed deaths. In the Southeast, Ebonyi State reported 40 suspected cases, 9 confirmed cases, and 4 deaths related to Lassa fever. Additionally, in the North Central zone, Kogi State reported 15 suspected cases, 5 confirmed cases, 1 probable case, and 3 deaths (3) (See Table 1).

**Table 1:** Cumulative reported cases and deaths of Lassa fever from January 6 to 26, 2025 (3) and compiled by Stanley Chinedu Eneh.

Affected state(s)	Cases			Deaths (confirmed cases)
	Suspected	Confirmed	Probable	
Ondo	328	107		10
Edo	358	61		10
Bauchi	205	49		5
Taraba	104	48		15
Ebonyi	40	9		4
Gombe	18	5		4
Kogi	15	5	1	2
Plateau	8	3		1
Nasarawa	32	2		2
Delta	4	1		
Benue	17			
Rivers	3			
Adamawa	3			
Ogun	2			
Abia	1			
Imo	3			
Bayelsa	1			
Oyo	5			
FCT	3			
Lagos	2			
Kano	2			
Kaduna	6			
Enugu	4			
Cross River	5			
Anambra	4			

### Current efforts to mitigate the outbreak of Lassa fever in Nigeria

The NCDC, in collaboration with various non-governmental organizations, is conducting an orientation on the use of the Lassa fever Advocacy Toolkit for preparedness and response. Additionally, plans are underway to host the 6th Lassa Fever Webinar Series in partnership with the Federal Ministry of Environment (FMENV) and other stakeholders. Healthcare workers (HCWs) in Bauchi, Ebonyi, and Benue states have been trained on case management as part of these initiatives (3).

In response to the resurgence of Lassa fever, the Nigeria Centre for Disease Control (NCDC) implemented several surveillance systems, which include the establishment of state-level emergency operation centers, the development of real-time reporting mechanisms, and the integration of laboratory networks to facilitate faster diagnoses (10). These measures aim to enhance the accuracy of outbreak detection, improve timeliness, and enable more effective responses.

In response to this prevailing situation, Doctors Without Borders (Médecins Sans Frontières—MSF) has been actively involved in providing support to the state healthcare system (11). Their interventions include constructing six health centers and providing comprehensive support for Lassa fever management (11). Plans are underway to establish specialist hospitals in each of the state’s three senatorial zones to improve access to quality healthcare, particularly in rural areas (12).

Furthermore, the Ebonyi state 2025 budget includes provisions aimed at strengthening public health infrastructure, surveillance systems, and community engagement to effectively prevent and control future outbreaks of Lassa fever and other communicable diseases. The state governor emphasized that in anticipation of the outbreak season, essential medical supplies, infection prevention and control materials, as well as laboratory diagnostic tools, had been distributed across the state, alongside the revitalisation of 171 primary healthcare centres (13).

At the national level, the NCDC had activated the Emergency Operations Centre (EOC) for Lassa fever, with the risk assessment indicating a high level, and emergency Response Level two was advised and activated (14). The EOC was activated to ensure seamless coordination of Lassa fever control and management activities using a One Health approach. In furtherance, the number of Lassa fever testing laboratories had increased from around nine to 13, with plans for further upgrades (15). Community engagement plays a vital role in controlling Lassa fever. Effective risk communication strategies, community education programs, and involving traditional leaders help raise awareness and promote preventive measures. Implementing rodent control measures at the community level also reduces transmission risks (16).

### Economic challenges and healthcare system strain in Nigeria

Nigeria has faced significant economic challenges, which have severely impacted the healthcare system’s ability to effectively manage public health emergencies like the current Lassa fever outbreak. These challenges are characterized by broader economic constraints, inadequate infrastructure, a shortage of skilled personnel, limited resources, and insufficient medical supplies. For Example, in Ebonyi state specifically, isolation centres, diagnostic tools, and trained personnel are insufficient to address the surge in cases effectively (17).

The agrarian economies of the most affected states, amidst these challenges, are particularly vulnerable to zoonotic diseases like Lassa fever. Limited public health investments have left these states ill-equipped to manage outbreaks effectively. Their heavy reliance on federal allocations, coupled with low internally generated revenue, restricts their ability to allocate sufficient resources for healthcare improvements and public health interventions (18, 19). The northern regions of Nigeria, which have been among the country’s poorest, face economic difficulties that undermine preventive measures. These economic challenges have contributed to other outbreaks, such as the cholera crisis (20).

According to Aloke (21), Lassa fever primarily affects individuals in the most productive years; hence, this outbreak has further strained the economy, reduced workforce productivity, and disrupted livelihoods, leading to increased absenteeism at work and economic stagnation. The financial burden on families, resulting from medical expenses and loss of income, perpetuates poverty and hinders economic recovery (22).

Reports have shown that the transition of Lassa fever intervention efforts from Médecins Sans Frontières to the Ebonyi State government in December 2024 has further exposed these vulnerabilities, necessitating strategic planning and funding for sustained progress (23). Thus, the current economic situation in Nigeria could undermine the current effort to mitigate the Lassa fever outbreak in Nigeria.

### Lassa fever drivers and contributing factors to the current outbreak

#### Climate change and environmental factors

The resurgence of Lassa fever is influenced by changes in climate, such as shifts in temperature and precipitation, which have disrupted the natural habitats and migration patterns of *Mastomys natalensis*, the primary rodent reservoir of the virus (24). As a result, these rodents are increasingly encroaching on human settlements, particularly in Nigeria’s middle-belt region. Prolonged droughts and unpredictable rainfall patterns force rodents from rural areas into urban centers, raising the risk of transmission between animals and humans (25).

### *Mastomys natalensis* reservoirs and human interface

Deforestation for agriculture and peri-urban expansion have pushed *Mastomys* colonies closer to human dwellings, increasing nightly contact with household food and waste (26).

Studies have identified that 47.2% (17 out of 36) of Nigerian states are highly endemic for haemorrhagic fevers such as Lassa fever (27). This widespread occurrence has been largely attributed to increased rodent infestation, particularly by rats. These rodents pose significant health risks to humans through direct contact—including scratches, bites, or contamination of open wounds, eyes, and mouth—as well as through the ingestion of food or water tainted with rat fur and fecal matter (28, 29). Moreover, rats are known carriers of *Leptospira* bacteria, which they shed into the environment via their fur, contaminating both soil and water sources (30). Ilori et al. (9) further reported high fatality rates linked to rat infestations in certain Nigerian states, particularly Edo, Ondo, and Ebonyi, which accounted for over 75% of confirmed Lassa fever cases, with respective mortality rates of 14.6, 24.2, and 23.4%.

### Lack of vaccine for human use

Nigeria has experienced recurring and substantial outbreaks of Lassa fever in recent years, a trend that has persisted into 2025 (1–3). The continued high burden of the disease across Africa highlights the urgent need for effective preventive and therapeutic strategies against Lassa fever. At present, no licensed treatments or antiviral drugs are specifically approved for Lassa fever. However, patients have been managed for decades using off-label options such as ribavirin and convalescent plasma therapy, despite limited clinical evidence supporting their efficacy (31).

#### Socioeconomic and behavioral factors

Socioeconomic and Behavioral factors play substantial roles in the spread of Lassa fever. Widespread poverty and substandard housing is an ideal environment for rodent infestations, thereby increasing the risk of disease transmission. In rural areas, traditional food storage methods often create a passage for rodents to access food supplies, facilitating the spread of the disease (32). On the other hand, urbanization has led to overcrowding, poor sanitation, and inadequate waste management, which further exacerbate the risk of outbreaks (32). Other cultural practices, such as bushmeat consumption, also contribute to the transmission of zoonotic diseases like Lassa fever. These interconnected factors underscore the need for a multifaceted approach to address the root causes of Lassa fever outbreaks in Nigeria.

#### Healthcare system challenges

Nigeria’s healthcare system faces significant challenges due to resource constraints, which have hindered efforts to control Lassa fever outbreaks. Limited funding severely impacts disease surveillance, delaying response times and allowing the virus to spread unchecked (33, 34). Diagnostic limitations, including a shortage of diagnostic facilities, hinder timely case confirmation, while the high cost of treatment, with ribavirin courses costing between $500 and $800, restricts access to care, particularly affecting low-income households. Inadequate training for healthcare workers on case management and infection prevention and control strategies further complicates the situation, leading to increased transmission within healthcare settings (35). Furthermore, weak political commitment, particularly at the sub-national level, impedes sustained investments in epidemic preparedness and undermines the implementation of national strategies at the local level. Budget allocations for Lassa fever control often depend on external donor funding and emergency responses rather than proactive planning. Coordination across different tiers of government remains fragmented, and workforce attrition due to brain drain and poor working conditions further compounds the healthcare system’s limited capacity (36).

### Gaps and challenges in disease control in Nigeria

Eradication of Lassa fever has faced various challenges and gaps in Nigeria. These have led to increased endemicity and have imposed a substantial burden on the public health system. One of such challenges is the underfunding of the health systems. Health system strengthening is often overlooked ahead of other sectors, which has led to the underdevelopment of disease surveillance and health systems. This has prompted most funding efforts toward epidemics to come from International Health Organizations (37). In the long term, this widens the control of epidemics as it makes the country become reactive rather proactive.

Lassa fever is more prevalent in rural areas; yet Nigeria’s healthcare delivery system is weakest in these areas (37). Rural communities are associated with poorer health outcomes, higher mortality rates, and lower life expectancy due to inadequate access to healthcare services (38). This increases the impact of the disease in these areas as they are characterized by poor access roads and unreliable internet connections. These lead also to limited access to health support and transmission of critical data. These deficiencies consequently lead to increased health and economic burden.

Another area of challenge is the misinformation and stigmatization that arises during the control of Lassa fever. Studies have shown that survivors of Lassa fever often face stigmatization (39, 40) which can discourage others from getting medical help when symptoms arise. This action can often result in delayed detection, diagnosis and treatment of Lassa fever. Moreso, as often seen in epidemics, there can be various misconceptions toward the disease such as modes of transmission and lack of trust in medical interventions contributing to the spread of the disease (1). These misconceptions can lead to a lack of follow up or utilisation of preventive measures for the disease among affected individuals. These persistent challenges highlight the need for effective strategies and approaches that can strengthen Nigeria’s response to Lassa fever. Lessons drawn from neighboring countries that have faced similar epidemics could offer valuable insights for bridging these gaps.

### Comparative lessons from Sierra Leone

Comparative experiences from countries such as Sierra Leone provide important lessons for Nigeria. In response to its own Lassa fever burden, Sierra Leone has implemented more centralized and coordinated control efforts, including the establishment of specialized Lassa fever treatment centers, robust case surveillance mechanisms, and international collaborations that ensure consistent funding and technical support (41). These initiatives, supported by political will and donor partnerships, have enabled quicker outbreak responses and improved health outcomes (42). Nigeria could adapt similar approaches by strengthening institutional coordination, decentralizing treatment access, and prioritizing long-term investments in outbreak preparedness. Additionally, implementing regular After-Action Reviews and engaging communities in prevention efforts can also improve Nigeria’s capacity to manage and control Lassa fever outbreaks effectively.

### Strategic recommendations and approaches

#### Strengthening laboratory infrastructure

Robust healthcare systems are the bedrock of epidemic preparedness and response. Priority should be given to upgrading diagnostic capacity through state-level laboratory development, expanding the NCDC molecular lab network, and improving the national reference laboratory. Investments in communication infrastructure for rapid public health messaging and capacity-building programs for epidemiologists, biostatisticians, and frontline responders will ensure system resilience during outbreaks (43).

#### Improving disease surveillance and utilisation of environmental data

Effective surveillance underpins early outbreak detection and containment. The Integrated Disease Surveillance and Response (IDSR) strategy must be strengthened, particularly at the Local Government Area (LGA) level. Health workers should also be trained to utilize ecological and meteorological data to anticipate Lassa fever trends, especially since the 2018–2019 spikes were likely linked to environmental factors (44). This can inform seasonal risk forecasts and guide targeted interventions.

#### Community-based interventions and education

Nationwide enlightenment campaigns must be scaled up using mass media and social media platforms. Health officials, community leaders, and the media must collaborate to build awareness and drive behavioral change, such as prompt case reporting and improved sanitation practices (45–47). Engaging the public as informed partners strengthens the frontline defense against outbreaks.

#### Investing in research and vaccine development

Despite the lack of an approved vaccine for the management of Lassa fever, immunization is the most practical control method. A small number of vaccine candidates, such as those based on the measles virus or adenovirus vectors, have advanced to the clinical testing stage; the majority are still in the preclinical or early stages of clinical development (43). Government agencies and business organizations must fund ongoing research to advance the development of vaccines.

#### Environmental and rodent control

To mitigate the risk of Lassa fever transmission, especially in Nigeria’s highly endemic regions, it is imperative to prioritize environmental and rodent control as a cornerstone of prevention. Strengthening the structural integrity of homes should be a national priority—specifically, sealing all holes, cracks, and potential rodent entry points to reduce infestation and breeding within human habitations.

In parallel, the strategic and safe deployment of rodenticides remains a crucial intervention for reducing rodent populations in both residential and communal areas (48). Given the persistent absence of a licensed vaccine for Lassa fever and the recurrent outbreaks reported in Nigeria, integrated rodent control must be complemented by targeted public education to promote behavioral modifications that reduce human-rodent contact (49, 50). These recommendations underscore the urgent need for a coordinated, community-based approach that combines environmental management, behavioral change communication, and local capacity building to reduce the burden of Lassa fever.

#### Cross-sectoral collaboration and policy

Strengthening cross-sectoral collaboration and coherent policy implementation is critical to effectively combat Lassa fever in Nigeria. Given the zoonotic nature of the disease—transmitted from rodents to humans—control efforts must transcend the health sector and engage environmental, agricultural, housing, and education sectors under a unified One Health approach (51).

At the national level, coordinated action is needed between the Nigeria Centre for Disease Control (NCDC), state ministries of health, environmental agencies, and local government authorities to establish integrated surveillance, response, and risk communication systems. These efforts should include community-level engagement to improve awareness, reporting, and early action.

Regionally, Nigeria must also align its response with the International Health Regulations (IHR 2005), which mandate the timely sharing of public health information through designated IHR National Focal Points. Sharing data with neighbouring countries and regional health bodies can facilitate quicker containment and cross-border preparedness (52).

## Conclusion

The persistent burden of Lassa fever in Nigeria reveals deep systemic vulnerabilities in public health preparedness, disease surveillance, and healthcare delivery. While previous responses have focused largely on emergency containment, a paradigm shift toward long-term, preventive strategies is essential. This review highlights that controlling Lassa fever requires more than technical solutions, it demands political will, sustained financing, and a multisectoral strategy grounded in community resilience and infrastructure development. Our findings contribute to the broader field of infectious disease management by reinforcing the value of integrated approaches, linking environmental health, socioeconomic drivers, and digital technologies. Moving forward, Nigeria must prioritize scalable interventions such as community-based surveillance, improved sanitation, and decentralized diagnostic capacity. If embedded into national policy and backed by sufficient resources, these actions can substantially reduce the burden of Lassa fever and set a precedent for tackling other endemic zoonotic diseases in West Africa.

## Data Availability

The original contributions presented in the study are included in the article/supplementary material, further inquiries can be directed to the corresponding author.
